# Utilizing surrogate endpoints in clinical research: a strategic approach to overcome practical challenges

**DOI:** 10.36416/1806-3756/e20250476

**Published:** 2026-03-05

**Authors:** Ignacio Esteban, Cecilia María Patino, Juliana Carvalho Ferreira

**Affiliations:** 1. Methods in Epidemiologic, Clinical, and Operations Research-MECOR-program, American Thoracic Society/Asociación Latinoamericana del Tórax, Montevideo, Uruguay.; 2. Gavi, the Vaccine Alliance, 1218 Geneva, Switzerland.; 3. Department of Preventive Medicine, Keck School of Medicine, University of Southern California, Los Angeles (CA) USA.; 4. Divisão de Pneumologia, Instituto do Coração, Hospital das Clínicas Faculdade de Medicina, Universidade de São Paulo, São Paulo (SP) Brasil.

## PRACTICAL SCENARIO

Group B streptococcus (GBS) is a major cause of neonatal morbidity and mortality, particularly in low- and middle-income countries. Several maternal GBS vaccine candidates are in advanced stages of development to protect infants from invasive disease. Conducting traditional efficacy trials, however, is challenging due to the low incidence of GBS, which requires large sample sizes, long follow-up periods, and substantial resources. As an alternative, a phase 3 trial has recently begun exploring a regulatory pathway based on safety and immunological endpoints, such as the serological threshold of risk reduction (SToRR), which may serve as a predictor of clinical benefit.^1^ Utilizing such surrogate outcome could enable earlier evaluation and approval of GBS vaccines, potentially reducing delays in access for high-burden populations.

## SURROGATE ENDPOINTS: DEFINITION AND RATIONALE

Surrogate endpoints are biomarkers or intermediate outcomes that substitute for direct measures of how a patient feels, functions, or survives. They are employed when patient-centered endpoints are difficult to measure due to rarity, long follow-up, ethical constraints, high costs, or logistical challenges. In vaccine trials, immunological markers such as antibody titers often serve as surrogates, under the assumption that they correlate with protection against disease. In other fields, measures such as FEV_1_ are used as surrogates for COPD severity.

The use of surrogate endpoints is particularly valuable when large-scale, long-term trials are impractical, as they offer early indicators of efficacy, accelerating the assessment process. However, reliance on surrogates can introduce uncertainty regarding the true clinical benefit and may fail to capture potential harms adequately. Chart 1 summarizes the main advantages, challenges, and limitations of surrogate outcomes in clinical research.

**Chart 1:** Surrogate outcomes in clinical research: key advantages, challenges, and limitations.

ADVANTAGES	
Accelerated development timelines	Surrogate endpoints allow early measurement of immune responses, enabling faster preliminary assessment of vaccine efficacy without waiting for clinical disease outcomes
Reduced sample size requirements	Fewer participants may be needed to detect a significant effect, reducing recruitment challenges and associated costs
Cost-effectiveness	Shorter study durations and smaller sample sizes lower overall trial costs, making vaccine development more feasible, especially in resource-limited settings
Ethical considerations	Early assessment of efficacy reduces participants’ exposure to potential risk compared with waiting for clinical endpoints
CHALLENGES AND LIMITATIONS	
Predictive validity	Surrogates may fail to predict true clinical outcomes or intervention effects, leading to uncertainty and misestimation of efficacy
Regulatory acceptance	Agencies require robust proof of surrogate reliability, often necessitating complex and time-consuming validation studies
Generalizability	Validation can be resource-intensive, sometimes impossible, and context-specific, limiting generalizability across populations, interventions, or disease stages
Safety information	Surrogates may provide limited or misleading safety data, increasing the risk of approving harmful interventions
Statistical and study designs	Small study effects, biases, and reduced sample size or follow-up can further compromise interpretation and trial reliability
Interaction of limitations	Predictive failure, safety gaps, bias, and context-specificity can combine to increase uncertainty and complicate risk-benefit assessment

## STATISTICAL CONSIDERATIONS

Proper statistical planning and validation are essential when using surrogate endpoints. The choice of a surrogate endpoint should consider biologic plausibility, ensuring a scientifically grounded link between the surrogate and the clinical endpoint; epidemiologic evidence from observational studies or prior trials supporting that relationship; analytical validation, confirming the consistency and strength of the association; and the surrogate threshold effect, when specific changes in the surrogate correspond to meaningful changes in the clinical outcome. Accurate sample size calculations are also important to ensure sufficient power, and proper handling of missing data is necessary to avoid bias and maintain reliability. Phase IV studies are typically required to confirm real-world effectiveness, as is expected for GBS vaccines following potential licensure from our practical scenario. The CONSORT-Surrogate extension provides recommended reporting standards for trials using surrogate endpoints as primary outcomes.^2^

## CHALLENGES AND LIMITATIONS

The use of surrogate endpoints in clinical research presents several challenges.^3^ They must be rigorously validated, as failure to predict true clinical outcomes or intervention effects can create uncertainty, over- or underestimate efficacy, which may lead to incorrect associations and potential implementation of harmful interventions. Validation is often resource-intensive, sometimes difficult, and context-specific, which can limit generalizability across populations, disease stages, interventions, or prior treatments. Furthermore, these limitations can interact; predictive failure, safety gaps, biases, small study effects, and reduced sample size or follow-up may combine to increase uncertainty and complicate interpretation and overall risk-benefit assessment. For the approval of new drugs, regulatory acceptance can also be challenging, as robust evidence is required to demonstrate that the surrogate reliably predicts clinical benefit.

## KEY POINTS

- Surrogate endpoints are indirect measures of clinical outcomes that enable more efficient clinical trials, reducing sample sizes, costs, and development timelines.
- Surrogate endpoints provide early indicators of efficacy, offering a practical solution when measuring clinical outcomes directly is difficult, but their use requires careful validation and analysis.
- Surrogate markers must be rigorously validated to ensure they reliably predict true clinical benefit.
- Proper statistical planning, including sample size calculations and handling of missing data, is critical to maintain trial reliability.
- Phase IV studies are necessary to confirm the real-world efficacy and safety of new interventions approved based on surrogate endpoints.
